# Fast track hip fracture care and mortality – an observational study of 2230 patients

**DOI:** 10.1186/s12891-019-2637-6

**Published:** 2019-05-24

**Authors:** Christian Thomas Pollmann, Jan Harald Røtterud, Jan-Erik Gjertsen, Fredrik Andreas Dahl, Olav Lenvik, Asbjørn Årøen

**Affiliations:** 1 Department of Orthopaedic Surgery, Akershus University Hospital, Lørenskog, Norway; 2 Institute of Clinical Medicine, Campus Ahus, University of Oslo, Oslo, Norway; 3 Norwegian Hip Fracture Register, Department of Orthopaedic Surgery, Haukeland University Hospital, Bergen, Norway; 4 Department of Clinical Medicine (K1), University of Bergen, Bergen, Norway; 5 Health Services Research Unit, Akershus University Hospital, Lørenskog, Norway; 6 Department of Data and Analytics, Akershus University Hospital, Lørenskog, Norway; 7 Department of Sports Medicine, Norwegian School of Sport Sciences, Oslo, Norway

**Keywords:** Hip fracture, Fast track, Mortality, Reoperation, Surgical site infection, Admission time, Time to surgery, Length of stay, Readmission, Norwegian hip fracture register

## Abstract

**Background:**

Hip fracture patients are frail and have a high mortality. We investigated whether the introduction of fast track care reduced the 30-day mortality after hip fractures.

**Methods:**

Fast track hip fracture care was established at our institution in October 2013. Data from the Norwegian Hip Fracture Register and electronic hospital records were merged for 2230 hip fracture patients operated in our department from January 2012 through December 2015. 1090 of these patients were operated before (conventional treatment group) and 1140 patients were operated after the introduction of fast track care (fast track group). Data were analysed by univariate analysis and binary logistic regression.

**Results:**

Mortality did not differ significantly between the conventional treatment group and the fast track group at 30 days (7.9% vs. 6.5%), 90 days (13.5% vs. 12.5%) and one year (22.8% vs. 22.8%). Median admission time and time to surgery were significantly shorter in the fast track group than in the conventional treatment group (1.1 h vs. 3.9 h and 23.6 h vs. 25.7 h, both *p* <  0.0001). The 30-day reoperation rate was significantly lower in the fast track group compared to the conventional treatment group (odds ratio = 0.35 (95% CI: 0.15–0.84), *p* = 0.019). A composite 30-day outcome (reoperation, surgical site infection and/or death) was significantly less frequent in the fast track group (8.1%) than in the conventional treatment group (10.7%) in unadjusted analysis (*p* = 0.006), but not after adjusting for age, gender, cognitive impairment and ASA score (odds ratio = 0.85 (95% CI: 0.63–1.16), *p* = 0.31, 8.0% missing). Reoperations within 1 year, surgical site infections, 30-day readmissions and length of hospital stay did not differ significantly between the conventional treatment group and the fast track group.

**Conclusions:**

Fast track hip fracture care is safe. However, we observed no statistically significant change in 30-day, 90-day or 1-year mortality after the introduction of fast track hip fracture care.

**Trial registration:**

The study was registered retrospectively at ClinicalTrials.gov (Protocol Record 284907) 6 December 2016.

## Background

Hip fracture patients represent one of the largest groups of patients in orthopaedic surgery. A hip fracture constitutes a serious injury for these typically frail and elderly patients. This is reflected in several studies reporting high mortality rates between 6 and 11% within 30 days [1–4] and between 20 and 30% within 1 year [2, 5, 6]. In addition to the individual fate, hip fractures pose a growing public health problem. Due to the increasing age of the population in the western world the hip fracture burden is predicted to increase substantially over the next decades [7].

Traditionally, orthopaedic research has focused on surgical techniques for the treatment of hip fractures [8, 9]. However, excess mortality after a hip fracture remains high [5, 10]. Therefore, a new approach is warranted to try to reduce the high mortality. One such approach is the development of standardized fast track care systems for hip fracture patients.

Fast track methodology refers to a comprehensive treatment concept for surgical patients which takes into account the patients’ co-morbidities, cognitive impairment and polypharmacy, and which focuses on stress reduction, opioid sparing pain relief, nutrition and early mobilization to promote postoperative recovery [11]. Fast track patient care was initially developed for elective abdominal surgery [11], but the principles have subsequently been applied to elective orthopaedic surgery with good results [12]. More recently, fast track care systems have also been reported for hip fracture patients [13–17]. However, the effect on mortality is unclear. While one study reported lower 1-year mortality in community dwellers [18] several other studies found no effect of fast track care on mortality in hip fracture patients [14–17, 19].

The primary aim of this study was to investigate if the introduction of fast track care at our institution reduced the 30-day mortality rate after hip fracture surgery. Secondary outcome measures were 90-day and 1-year mortality, any cause reoperation, surgical site infection, a composite 30-day outcome (reoperation, surgical site infection or death), admission time, time to surgery, length of hospital stay and 30-day readmission.

## Methods

This study is reported according to the REporting of studies Conducted using Observational Routinely-collected health Data (RECORD) Statement [20].

### Conventional hip fracture care

Patients with suspected hip fracture were admitted via the accident and emergency (A&E) department, Akershus university hospital (AHUS). The patients had to wait for an available examination room, an available physician and a slot in the radiology lab. After x-ray examination the patients were transported back to the A&E department where the admitting physician evaluated the x-rays and finished the work-up before the patient was transported to the orthopaedic ward.

Apart from antithrombotic and perioperative antibiotic prophylaxis, perioperative treatment was not standardized. There was no systematic focus on nutritional status and on prevention and treatment of delirium. The patients were not consistently mobilized on the first postoperative day.

### Fast track hip fracture care

Fast track hip fracture care was introduced at AHUS in two steps. From 28 October 2013, guidelines concerning perioperative treatment were taken into practice. Secondly, 27 January 2014, the fast track pathway for hospital admission was implemented.

The ambulance personnel initiate first line treatment (intravenous fluids, oxygen, pain relief and electrocardiogram). Upon arrival at the A&E department a trained nurse triages all suspected hip fracture patients using the Manchester triage system [21]. If deemed necessary, additional intravenous opiates are administered in selected cases.

Patients without signs of other, more acute medical conditions (score of 3 or higher) continue in the fast track admission pathway if they fulfil the following criteria: Low energy trauma, hip/groin pain, shortened and/or externally rotated lower extremity and/or unable to bear weight, no sign of other fractures, no sign of neurovascular injury, not previously operated on the hip in question.

From the triage area the patient is brought directly to the radiology lab where fast track hip fracture patients are prioritized after any ongoing procedure.

The radiology technician evaluates the x-ray. If considered to have a hip fracture the patient is transported directly to the orthopaedic ward where a nurse performs standard procedures according to a check-list and gives the patient both oral and written information about hip fractures and the expected course of treatment.

The orthopaedic surgeon re-evaluates the x-ray, writes an admission note, administers a fascia iliaca compartment block and prescribes a set of standard medications, including oral and intravenous fluids and pain medication.

The fast track hip fracture care system includes written guidelines concerning standard blood sampling, premedication, pre- and postoperative pain relief with focus on opiate sparing, pre- and postoperative fluid treatment with focus on short periods of fasting, transfusion-triggers and management of anticoagulants. Patients are mobilized on the first postoperative day. The guidelines also advise on screening for and prevention and treatment of delirium, on screening of nutritional status and on appropriate interventions concerning nutrition.

### Data collection

All primary and revision hip fracture operations in Norway should be reported to the Norwegian Hip Fracture Register (NHFR) [22]. This is done prospectively by the surgeon on a 1-page questionnaire which includes information on the type of fracture, American Society of Anaesthesiologists (ASA) score [23], cognitive impairment (possible choices: ‘no’, ‘uncertain’, ‘yes’), type of anaesthesia, type of operation, surgeon’s experience (at least one surgeon with > 3 years of experience in hip fracture surgery) and operating time (time from incision to skin closure).

We obtained the NHFR data for patients operated at AHUS from January 2012 through December 2015. For the same time period, hip fracture patients were identified from the electronic hospital records using the search strings main diagnosis S72.0, S72.1 or S72.2 (ICD-10), in-patient, operated during that hospital admission. The two databases were linked deterministically using the unique 11-digit Norwegian personal identification number. For patients who were only identified in one of the data sources or for whom the records did not match, the electronic hospital records were scrutinized to determine if there had occurred an error in coding or in reporting to the NHFR.

### Patients

**Fig. 1:**
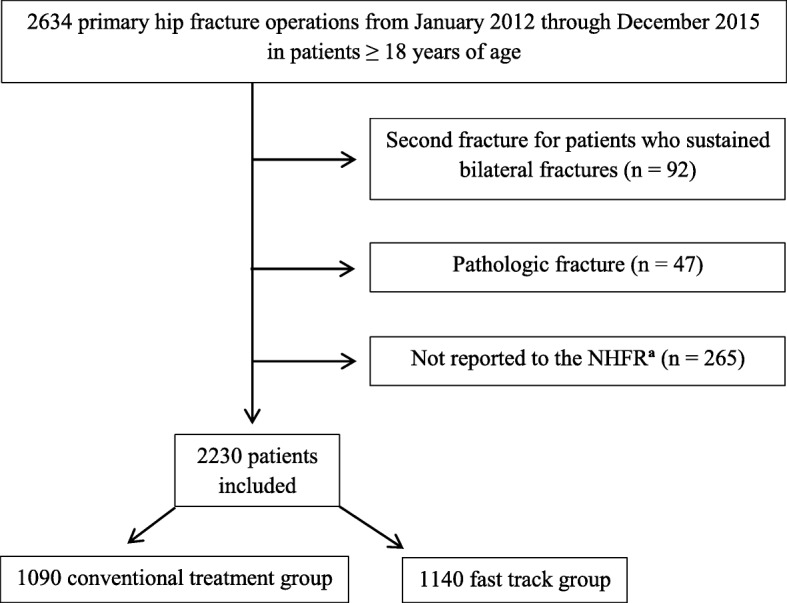
Flow chart of patient inclusion. ^a^Norwegian Hip Fracture Register

All patients 18 years of age or older who were operated for a fracture of the proximal femur (femoral neck, trochanteric or subtrochanteric) at a single institution (AHUS) from January 2012 through December 2015 were eligible for inclusion (Fig. 1). AHUS has a catchment area of approximately 500.000 inhabitants.

During the study period 2634 primary hip fracture operations were performed. For patients who sustained two hip fractures during the study period (*n* = 92), only the first fracture was included in the analysis. Patients with a pathologic fracture were excluded (*n* = 47). 265 patients were not reported to the NHFR, corresponding to an overall reporting rate from AHUS of 89.4% (87.9% before and 90.8% after the introduction of fast track hip fracture care). The remaining 2230 patients, who were reported to the NHFR, represent the study population. Of these 1090 patients were operated before (conventional treatment group) and 1140 patients were operated after the introduction of fast track hip fracture care (fast track group) with 28 October 2013 as cut-off. The available data do not allow us to determine to what extent the different components of the reported fast track care system were applied to an individual patient. Therefore, the analysis follows the intention to treat principle and hip fracture patients treated from 28 October 2013 and onwards are included in the fast track group, irrespective of length of admission time or other criteria.

### Operative treatment

Patients with a femoral neck fracture were treated either with closed reduction and internal fixation with two screws, with a cemented bipolar hemiarthroplasty or with a cemented total hip arthroplasty, both with a taper slip stem using a direct lateral approach. Trochanteric fractures were treated with a sliding hip screw and subtrochanteric fractures with an intramedullary nail. Surgical treatment guidelines did not differ before and after the introduction of fast track hip fracture care.

### Outcome measures

#### Mortality

Mortality data from the Central Population Register are routinely imported to the electronic hospital records and a last up-date of the database was performed on 14 September 2017 to allow for a delay in registration. There was no loss to follow-up. 30-day, 90-day and 1-year mortality were calculated from time of arrival at the hospital. Survival was censored at 1 year.

#### Reoperations

Data on reoperations were obtained from the NHFR. Time to event was calculated from the index operation. In 2013 and 2014 the reporting rate from our institution was 66% for reoperations after osteosynthesis and 81.6% for reoperations after hemiarthroplasty [24].

#### Surgical site infection

The Department of Microbiology and Infection Control, AHUS, surveys surgical site infections after hemiarthroplasty and total arthroplasty of the hip under the Norwegian Surveillance System for Antibiotic Use and Hospital-Acquired Infections (NOIS) [25] with 30-day and one-year follow-up. Sliding hip screws are not monitored by NOIS, but the Department of Microbiology and Infection Control, AHUS, surveys surgical site infection also in these patients with a 30-day follow-up using the same criteria. The completeness of the 30-day follow-up ranged from 97.7 to 99.6% per calendar year. The completeness of the 1-year follow-up ranged from 98.7 to 99.5% for hemiarthroplasties and from 87.6 to 91.9% for total hip arthroplasties.

Internal fixations of femoral neck fractures and intramedullary nails are not systematically surveyed for surgical site infection.

Time to event was calculated from the time of the index operation.

### Composite 30-day outcome

A patient was considered to have had this (negative) outcome if any of the following had occurred: death within 30-days from arrival at the hospital, reoperation or infection within 30-days from the index operation.

### Readmission

Readmission was defined as any cause, non-elective readmission within 30 days after discharge from the index admission. These data were extracted from the electronic hospital records.

### Admission time, time to surgery and length of hospital stay

Admission time (time from arrival at the hospital to arrival at the orthopaedic ward), time to surgery (time from arrival at the hospital to skin incision) and length of hospital stay were extracted from the electronic hospital records.

### Statistical analysis

A sample size calculation based on a reduction of the 30-day mortality rate after hip fracture from 10.7% (AHUS in 2011 [26]) to 6.8% (hospital with lowest mortality rate in Norway in 2011 [26]), 85% power and a level of significance of 0.05 yielded a total sample size of approximately 1800 patients [27].

Fisher’s exact test was used for unadjusted comparisons of proportions, while the Chi square test was used for unadjusted comparisons of ordinal and nominal distributions. Student’s T test was used for unadjusted comparisons of continuous variables. However, comparisons of admission times, time to surgery and length of hospital stay were made with non-parametric Mann-Whitney U tests, rather than t-tests, due to the skewed distributions of these variables.

Logistic regression was used to analyze the effects of different predictors on the binary outcomes of mortality at 30 days, 90 days and 1 year follow-up, reoperation and surgical site infections at 30 days and 1 year follow-up, as well as 30-day readmission and the composite 30-day outcome. The main predictor of interest in these models was the conventional treatment/fast track care indicator. Other variables were included as confounders if they showed statistical significance at the 0.05 level, except for patient age and gender, which were always included. All municipalities and Oslo districts belonging to AHUS’ catchment area were included in the analysis with a distinct identifier while patients from outside our hospital’s catchment area were coded as one group. This variable was considered as a random effect in the models, but turned out not to have a significant effect. Reducing time to surgery is one of the intended effects of fast track care. Therefore, the logistic regression model analyzing the effect of fast track care on mortality was run with and without including time to surgery as an independent predictor. This did not relevantly change the result for the effect of fast track care on mortality.

Survival analysis by Cox regression was considered for the binary outcomes, since these were all associated with event times. However, there were problems with the assumptions of proportional hazards, measured by Schoenfeld residuals ph-test. Concerning mortality there was no loss to follow-up, so the logistic regression models’ inability to handle right-censoring was not an issue. Also, the standard quality indicators of hip surgery are defined as the number of adverse outcomes after 30 days, 90 days and 1 year, which is in line with logistic regression. Logistic regression was therefore chosen.

A post hoc power analysis was performed using standard normal distribution approximation.

### Subgroup analyses

Patients were divided into two subgroups according to their comorbidity (ASA score). Frailer patients were defined by an ASA score ≥ 3.

## Results

Baseline characteristics for the conventional treatment group and the fast track group are shown in Table 1.

**Table 1:** Baseline characteristics

	Conventional treatment group (*n*^a^ = 1090)	Fast track group (*n*^a^ = 1140)	*p*-value^b^
Age^c^ (years)	79.7 (0.3)	79.6 (0.3)	0.69
Gender^d^			0.5
Women	740 (67.9)	789 (69.2)	
ASA^d^			0.002
ASA 1	36 (3.4)	27 (2.4)	
ASA 2	292 (27.3)	358 (32.1)	
ASA 3	609 (56.9)	641 (57.5)	
ASA 4	130 (12.1)	85 (7.6)	
ASA 5	3 (0.3)	4 (0.4)	
Missing	20 (1.8)	25 (2.2)	
Cognitive impairment^d^			0.041
No	679 (65.9)	737 (70.2)	
Uncertain	102 (9.9)	107 (10.2)	
Yes	249 (24.2)	206 (19.6)	
Missing	60 (5.5)	90 (7.9)	
Type of fracture^d^			0.69
Femoral neck, undisplaced	171 (15.7)	181 (15.9)	
Femoral neck, displaced	445 (40.9)	439 (38.6)	
Basocervical	31 (2.9)	25 (2.2)	
Trochanteric, 2 fragments	166 (15.3)	176 (15.5)	
Trochanteric, > 2 fragments	169 (15.5)	180 (15.8)	
Intertrochanteric	21 (1.9)	27 (2.4)	
Subtrochanteric	37 (3.4)	42 (3.7)	
Other	47 (4.3)	66 (5.8)	
Missing	3 (0.3)	4 (0.4)	

^a^Number of patients in group

^b^Test for equal distribution in both groups (Student’s T-test for age, Chi square test for all other parameters)

^c^Mean (standard error)

^d^n (%)

Data on surgical treatment for the conventional treatment group and the fast track group are shown in Table 2.

**Table 2:** Surgical treatment

	Conventional treatment group (*n*^a^ = 1090)	Fast track group (*n*^a^ = 1140)	*p*-value^b^
Type of operation^c^			0.25
Hemiarthroplasty	450 (41.3)	434 (38.1)	
Screw osteosynthesis	169 (15.5)	193 (16.9)	
Sliding hip screw	403 (37.0)	422 (37.0)	
Intramedullary nail	29 (2.7)	34 (3.0)	
Total hip replacement	38 (3.5)	57 (5.0)	
Resectionarthroplasty	1 (0.1)	0 (0)	
Missing	0 (0)	0 (0)	
Type of anesthesia^c^			0.002
Spinal	916 (88.6)	912 (85.5)	
General	74 (7.2)	122 (11.4)	
Other	44 (4.3)	33 (3.1)	
Missing	56 (5.1)	73 (6.4)	
Surgeon’s experience^c^			0.75
≤ 3 years	141 (14.3)	157 (14.9)	
> 3 years	842 (85.7)	900 (85.1)	
Missing	107 (9.8)	83 (7.3)	
Operating time^d^ (min.)	60 (1)	63 (1)	0.003

^a^Number of patients in group

^b^Test for equal distribution in both groups (Student’s T-test for operating time, Chi square test for all other parameters)

^c^n (%)

^d^Mean (standard error)

### Mortality

30-day, 90-day and 1-year mortality did not differ significantly between the conventional treatment group and the fast track group (Table 3). This was consistent in both unadjusted and adjusted analyses. In the adjusted analyses, age, male gender, cognitive impairment and increasing ASA score were independent predictors of increased mortality (Table 4) while fast track care, time to surgery, surgeon’s experience, type of fracture, type of operation, type of anaesthesia, operating time and municipality were not.

**Table 3:** Mortality

	Conventional treatment group (*n*^a^ = 1090)	Fast track group (*n*^a^ = 1140)	Between group difference
	% (95% CI ^b^)	% (95% CI ^b^)	% (95% CI ^b^)
30-day mortality	7.9 (6.4 to 9.7)	6.5 (5.1 to 8.1)	−1.4 (−3.7 to 0.9)
90-day mortality	13.5 (11.5 to 15.7)	12.5 (10.6 to 14.5)	−1.0 (−3.9 to 1.8)
1-year mortality	22.8 (20.4 to 25.5)	22.8 (20.4 to 25.4)	0 (− 3.6 to 3.5)

^a^Number of patients in group

^b^95% confidence interval

**Table 4:** Independent predictors of mortality

	30-day mortality		90-day mortality		1-year mortality	
	Odds ratio (95% CI^a^)	*p*-value	Odds ratio (95% CI^a^)	*p*-value	Odds ratio (95% CI^a^)	*p*-value
Age (years)	1.05 (1.03–1.07)	< 0.0001	1.05 (1.03–1.07)	< 0.0001	1.04 (1.03–1.05)	< 0.0001
Male gender	2.08 (1.45–2.98)	< 0.0001	1.91 (1.43–2.56)	< 0.0001	1.73 (1.40–2.19)	< 0.0001
Cognitive impairment uncertain	1.75 (1.03–2.96)	0.037	1.66 (1.09–2.54)	0.018	1.45 (1.02–2.06)	0.037
Cognitive impairment	2.86 (1.95–4.19)	< 0.0001	3.03 (2.24–4.09)	< 0.0001	2.56 (1.99–3.29)	< 0.0001
ASA^b^-score	3.44 (2.54–4.65)	< 0.0001	3.34 (2.61–4.26)	< 0.0001	3.09 (2.53–3.77)	< 0.0001

Logistic regression; 8.0% missing

^a^95% confidence interval

^b^American Society of Anaesthesiologists

Fast track hip fracture care had no significant effect on 30-day, 90-day or 1-year mortality in subgroup analyses of healthier (ASA score ≤ 2) and frailer (ASA score ≥ 3) patients.

**Fig. 2:**
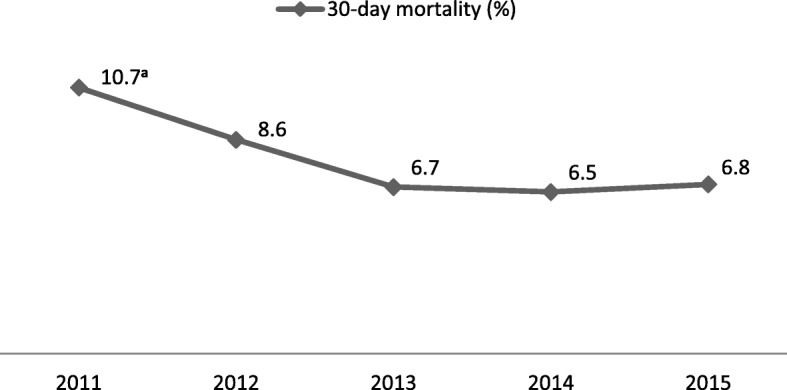
Decrease in 30-day mortality before introduction of fast track care. ^**a**^ from Helgeland J et al. 2013 [26]

30-day mortality was 10.7% in 2011 [26], before the start of the study. It decreased to 8.6% in 2012 and 6.7% in 2013 before the introduction of fast track care and remained stable in 2014 (6.5%) and 2015 (6.8%) (Fig. 2).

A post hoc power analysis, given the sample size and the observed 30-day mortality rate of 7.9% in the conventional treatment group, showed that the study had 80% power to detect a decrease of the 30-day mortality rate to 5.0% in the fast track group with a level of significance of 0.05.

### Secondary outcome measures

Secondary outcome measures for the conventional treatment group and the fast track group are presented in Table 5.

**Table 5:** Secondary outcome measures

	Conventional treatment group (*n*^a^ = 1090)	Fast track group (*n*^a^ = 1140)	Between group difference
	% (95% CI ^b^)	% (95% CI ^b^)	% (95% CI ^b^)
Any cause reoperation 30 days	1.7 (1.1 to 2.7)^¥^	0.6 (0.2 to 1.3)^¥^	−1.1 (− 2.2 to − 0.03)
Any cause reoperation 1 year	5.8 (4.5 to 7.3)	4.3 (3.2 to 5.6)	− 1.5 (− 3.4 to 0.5)
Surgical site infection 30 days^c^	2.5 (1.6 to 3.7)	1.8 (1.0 to 2.8)	−0.7 (− 2.2 to 0.7)
Surgical site infection 1 year^c, d^	3.0 (2.0 to 4.4)	2.0 (1.2 to 3.1)	−1.0 (− 2.7 to 0.5)
Composite 30-day outcome	10.7 (9.0 to 12.7)^#^	8.1 (6.6 to 9.8)^#^	−2.6 (−5.3 to −0.06)
30-day readmission	12.8 (10.9 to 15.0)	11.7 (9.9 to 13.7)	−1.1 (−4.0 to 1.6)

^a^Number of patients in group

^b^95% confidence interval

^c^Data available for hemiarthroplasty, total hip arthroplasty and sliding hip screws

^d^Sliding hip screws only followed up for infection for 30 days

^¥^Statistically significant difference in unadjusted (Fisher’s exact test, *p* = 0.017) and adjusted analysis (binary logistic regression) (odds ratio = 0.35 (95% CI: 0.15–0.84), *p* = 0.019, 0% missing)

^#^Statistically significant difference in unadjusted analysis (Fisher’s exact test, *p* = 0.006)

The 30-day reoperation rate was lower in the fast track group (0.6%) than in the conventional treatment group (1.7%) (*p* = 0.017). After adjusting for age and gender, fast track hip fracture care remained an independent predictor of a lower 30-day reoperation rate (OR = 0.35, (95% CI: 0.15–0.84), *p* = 0.019, 0% missing).

The composite 30-day outcome (reoperation, surgical site infection and/or death) was less frequent in the fast track group (8.1%) compared to the conventional treatment group (10.7%) in an unadjusted analysis (*p* = 0.035). However, after adjusting for age, gender, cognitive impairment and ASA score, the odds-ratio for fast track care was no longer statistically significant (OR = 0.85 (95% CI: 0.63–1.16), *p* = 0.31, 8.0% missing).

Reoperation within 1 year, surgical site infection and 30-day readmission did not differ significantly between the conventional treatment group and the fast track group.

### Admission time, time to surgery and length of hospital stay

The median time from arrival at the hospital to arrival at the orthopaedic ward (admission time) and from arrival at the hospital to the start of surgery was significantly shorter in the fast track group compared to the conventional treatment group while the median length of hospital stay did not differ significantly (Table 6).

**Table 6:** Admission time, time to surgery and length of hospital stay

	Conventional treatment group (*n*^a^ = 1090)		Fast track group (*n*^a^ = 1140)	
	n^b^	Median (IQ-range^c^)	n^b^	Median (IQ-range^c^)
Admission time (hours)	1053	3.9 (2.9–5.2)*	1061	1.1 (0.6–3.2)*
Time to surgery (hours)	1054	25.7 (18.9–39.7)^#^	1072	23.6 (18.0–32.6)^#^
Length of stay (days)	1054	5.3 (4.0–7.0)	1072	5.2 (4.0–7.3)

^a^Number of patients in group

^b^Number of patients with available data

^c^Interquartile range

*Statistically significant difference (Mann Whitney U test, *p* < 0.0001)

^#^Statistically significant difference (Mann Whitney U test, *p* < 0.0001)

## Discussion

Although the introduction of fast track hip fracture care significantly reduced admission time, time to surgery and the risk of reoperation within 30 days, we observed no significant change in 30-day, 90-day or 1 year mortality. The composite 30-day outcome (reoperation, surgical site infection and/or death) was significantly less frequent in the fast track group in univariate analysis. However, in multivariate analysis this difference was no longer significant. There was a numerical trend towards fewer reoperations within 1 year, fewer surgical site infections and fewer 30-day readmissions in the fast track group, but this was not statistically significant. The length of hospital stay did not differ significantly between the conventional treatment group and the fast track group.

Our study was observational, investigating the effect of introducing fast track care as a quality improvement measure. This entailed that the patients did not follow a rigorous study protocol. We do not know exactly how many patients were admitted via the fast track admission pathway, but the data on admission time would suggest that this was the case for only about half of the patients. Thus, the relatively high percentage of patients who were not “fast tracked” to the orthopaedic ward may have contributed to not finding a statistically significant effect of fast track care on mortality. However, the importance of admission time for postoperative outcome is still controversial with shorter admission time being associated with higher in-hospital mortality in one study [28] and with fewer postoperative complications in another [13]. The effect of preoperative waiting time on postoperative outcome is not unequivocal either [29]. However, an increasing body of evidence suggests that a longer time to surgery correlates with increased mortality [4, 30], risk of infection [31] and other complications [32]. Thus, the rather modest reduction in time to surgery of just over two hours in the fast track group compared to the conventional treatment group might have contributed to not finding a statistically significant effect of fast track care on mortality. However, time to surgery was not an independent predictor of mortality in our cohort.

Our sample size calculation was based on our institution’s 30-day mortality rate in 2011. However, 30-day mortality decreased considerably in 2012 and 2013, before fast track care was introduced, and subsequently levelled off. Thus, our sample size calculation was based on a higher mortality rate in the conventional treatment group than we did observe, which would have made it difficult to detect a possible effect of fast track care on mortality. What caused this improvement is unclear. The preparations to introduce fast track care started in 2012 and one could speculate that the increased focus on hip fracture patients may have had a positive effect already before fast track care was taken into practice. Another possible scenario is that the introduction of fast track care had a negative effect and interrupted a positive time trend of decreasing mortality. However, this seems less likely since the 30-day mortality levelled off at a value that lies in the lower range of reported rates [1–4].

One also has to consider the possibility that the continuous improvement of in-hospital hip fracture care has resulted in mortality rates for this frail group of patients which become increasingly difficult to reduce. This notion seems to be supported by the fact that also other recent approaches to improve hip fracture care, such as geriatric co-management, had mixed results with some studies reporting a statistically significant effect on mortality [33, 34] while other studies did not [35, 36]. A recent Cochrane review was not quite conclusive concerning mortality, but stated that comprehensive geriatric assessment probably reduces mortality in older people with hip fracture (risk ratio 0.85, 95% CI 0.68 to 1.05; 5 trials, 1316 participants, inconsistency (I^2^) = 0%; moderate-certainty evidence) [37]. While in-hospital care is undoubtedly a cornerstone of hip fracture treatment, improvements in rehabilitation in the primary health care sector might also be warranted [19].

Our findings are in agreement with several other studies of fast track care systems for hip fracture patients that found no effect on mortality [14–17, 19]. While Eriksson et al. [14], Larsson et al. [16] and Hansson et al. [19] focused on bypassing the A&E department to reduce time to surgery, Haugan et al. [17] reported on a more comprehensive fast track system, comparable to the one described in our study. Using a retrospective study design they compared a cohort of 788 hip fracture patients treated before to 1032 patients treated after the introduction of fast track care and found no difference in 30-day, 90-day or 1 year mortality. In contrast, Pedersen et al. [18], who retrospectively investigated a similar fast track system, found a significantly lower 1-year mortality rate in their fast track group (12 versus 23%) when looking at the subgroup of community dwelling patients. This reduction in mortality is quite pronounced and the reason for this apparent discrepancy with our and the above mentioned findings is unclear. The study by Pedersen et al. [18] was based on a retrospective chart review. However, the intervention group was defined by time period and mortality data was obtained from the Civil Registration Office leaving little room for error. Another conceivable explanation for a potentially spurious positive finding by Pedersen et al. may be their relatively small sample size. Their cohort comprised 553 patients of which 376 were community dwellers compared to a total of 1820 patients in the report by Haugan et al. [17] and 2230 patients in this paper. Information on prefracture living arrangements was not available in our study. However, we performed a subgroup analysis of healthier patients, who can be expected to live in the community, and found no statistically significant effect of fast track care on mortality.

The presented study has several strengths. This study is, to our knowledge, the largest to date investigating fast track care for hip fracture patients. There was no loss to follow-up concerning the main outcome measure of mortality. The wide inclusion criteria imply that the study population was representative and that the results thus can be generalized.

Furthermore, this study is based on high quality data. The NHFR records its data prospectively. In addition, the data from the NHFR were cross referenced with data from the electronic hospital records thereby further increasing the data quality.

The study also has limitations. Data from the electronic hospital records were acquired retrospectively. It is not possible to discriminate the effects of the different components of the described fast track care system. Data on admission time suggests that only about half of all patients in the fast track group were admitted via the fast track admission pathway. However, the expedient admission is only one of several components of a fast track hip fracture care system. There were small, but due to the large number of patients, statistically significant differences between the groups in several of the baseline characteristics. However, in the multivariate logistic regression analyses these differences were adjusted for. During the study period, 11% of all primary hip fracture operations at our institution were not reported to the NHFR. However, with a reporting rate of 87.9% before and 90.8% after the introduction of fast track hip fracture care we consider it reasonable to assume that the reporting practice remained largely unchanged throughout the study. The reporting rate of reoperations to the NHFR is inferior to the reporting rate of primary operations [24]. Nevertheless, there is no reason to believe that the reporting rate of reoperations changed during the study period. Thus, the crude number of reoperations is probably higher than reported in this study, but the risk differences between the groups of patients studied should not be influenced by under-reporting of reoperations in only one of the groups. While surgical site infections after hemiarthroplasty and total arthroplasty of the hip were followed up after 30 days and one year, sliding hip screws were only followed up after 30 days and internal fixations of femoral neck fractures and intramedullary nails were not followed up for this complication. However, this procedure specific difference in follow-up for surgical site infection applies equally to both the conventional treatment group and the fast track group.

Since this study is based on register data it is not possible to determine to what extent an individual patient received treatment according to the department’s fast track hip fracture care guidelines. However, while the inclusion in a clinical trial will in itself influence any outcome measure [38] this is not the case for this register based study. The presented data thus reflect the effect on mortality and the secondary outcome measures one can expect by introducing a fast track hip fracture care system similar to the one described as a quality improvement measure.

We observed no increase in complications or readmissions after the introduction of fast track care which seems to indicate that “fast tracking” hip fracture patients to the orthopaedic ward after triage by trained health care personnel is safe. Even though fast track care did not significantly change mortality in this study, there was a numerical trend to improvement for all outcome measures and fast track care for hip fracture patients is still in place at our institution. Efforts to further improve hip fracture care should probably focus on even shorter preoperative waiting times [4] in combination with a fast track care system, geriatric co-management [37] and intensified rehabilitation after hospital discharge [19].

## Conclusions

Fast track hip fracture care is safe. However, we observed no statistically significant change in 30-day, 90-day or 1-year mortality after the introduction of fast track hip fracture care.

## Abbreviations

95% CI: 95% confidence interval
A&E: Accident and emergency
AHUS: Akershus university hospital
ASA: American Society of Anaesthesiologists
IQ-range: Interquartile range
NHFR: Norwegian Hip Fracture Register
OR: Odds ratio
RECORD: REporting of studies Conducted using Observational Routinely-collected health Data Statement
